# *PHACTR1* Gene Polymorphism Is Associated with Increased Risk of Developing Premature Coronary Artery Disease in Mexican Population

**DOI:** 10.3390/ijerph13080803

**Published:** 2016-08-09

**Authors:** Nonanzit Pérez-Hernández, Gilberto Vargas-Alarcón, Rosalinda Posadas-Sánchez, Nancy Martínez-Rodríguez, Carlos Alfonso Tovilla-Zárate, Adrián Asael Rodríguez-Cortés, Oscar Pérez-Méndez, Ruben Blachman-Braun, José Manuel Rodríguez-Pérez

**Affiliations:** 1 Department of Molecular Biology, National Institute of Cardiology “Ignacio Chávez”, Juan Badiano No. 1, Col. Sección XVI, Tlalpan 14080, Mexico City, Mexico; unicanona@yahoo.com.mx (N.P.-H.); gvargas63@yahoo.com (G.V.-A.); asaelrodriguezcortes@hotmail.com (A.A.R.-C.); opmendez@yahoo.com (O.P.-M.); rubenblach@gmail.com (R.B.-B.); 2 Department of Endocrinology, National Institute of Cardiology “Ignacio Chávez”, Mexico City 14080, Mexico; rossy_posadas_s@yahoo.it; 3 Community Health Research Department, Children’s Hospital of Mexico “Federico Gomez”, Ministry of Health (SSA), Mexico City 06720, Mexico; amr70@hotmail.com; 4 Juarez Autonomous University of Tabasco, Multidisciplinary Academic Division of Comacalco, Villahermosa 86040, Mexico; alfonso_tovillasz@yahoo.com.mx

**Keywords:** coronary artery disease, genetic polymorphisms, vascular calcification, *PHACTR1* gene

## Abstract

Single-nucleotide polymorphisms (SNPs) in the protein phosphatase and actin regulator 1 gene (*PHACTR1*) have been associated with susceptibility to develop several diseases, including cardiovascular disease. The purpose of this study was to evaluate the role of two polymorphisms (rs2026458 and rs9349379) of the *PHACTR1* gene in the susceptibility to the risk of developing premature coronary artery disease (CAD) in the Mexican population. The genotype analysis was performed using 5’exonuclease TaqMan genotyping assays in a group of 994 patients with premature CAD and 703 controls. A similar genotype distribution of rs2026458 was observed in both groups; however, under an additive model adjusted by age, body mass index, type 2 diabetes mellitus, smoking, dyslipidemia, and hypertension, the rs9349379 G allele was associated with a higher risk for developing premature CAD (odds ratio (OR) = 1.22, 95% confidence interval (CI) = 1.03–1.46, *p*-value (*p*) = 0.024). The two *PHACTR1* polymorphisms were not in linkage disequilibrium. In summary, our results suggest that the *PHACTR1* rs9349379 polymorphism plays an important role in the risk of developing premature CAD in the Mexican population.

## 1. Introduction

Coronary artery disease (CAD) is a complex multifactorial and polygenic disease that has been associated with an excessive inflammatory response in the vascular wall [1,2]. In developed countries, CAD is a major public health problem; and it represents the main cause of death worldwide. It is well known that different risk factors, such as insulin resistance, diabetes mellitus, hypertension, obesity, dyslipidemia, alcohol consumption and smoking, are associated with the development and progression of the atherosclerotic plaques [3]. Numerous genome-wide association studies (GWASs) performed in different ethnic groups have shown associations of several gene polymorphisms with cardiovascular disease [4,5,6,7]. Although the complete molecular pathways in which the genetic variants predispose people to cardiovascular disease are still unknown, recently several possible mechanisms have been proposed; for instance, it has been postulated that phosphatase regulation plays a specific role in CAD pathophysiology, through protein phosphatase and actin regulator 1 (PHACTR1), a molecule that inhibits protein phosphatase-1 (PP1) and binds actin via the C-terminal domain [8]. In addition, PP1 is an enzyme that dephosphorylates serine and threonine residues of several substrates and thus influences various cellular processes [9]. Moreover, different studies have analyzed and demonstrated the association of *PHACTR1* gene polymorphisms with susceptibility to CAD in different populations (e.g., European, Asian, and Lebanese) [5,10,11,12]. In addition, phosphatases have lately been associated with several diseases, such as cancer, diabetes mellitus, hypertension, and cardiovascular disease [13,14,15].

In the *PHACTR1* gene, two single-nucleotide polymorphisms (SNPs) have been associated with cardiovascular disease; the locations of these variants are 12825874 C > T (rs2026458) and 12903957 A > G (rs9349379). However, when we performed the haplotype analysis between these SNPs, we did not detect coefficient of linkage disequilibrium (D’ = 0.52; rate of recombination (r2) = 0.50). Interestingly, the gene expression analysis of *PHACTR1* revealed an association with transcription levels in human coronary arteries [16]. Large GWASs have shown an association of the rs2026458 polymorphism with coronary artery calcification [6]. In addition, the rs9349379 polymorphism has been associated with coronary artery stenosis [5], CAD [17] and coronary artery calcification [18]. To our knowledge, less than 15 reports have associated *PHACTR1* gene polymorphisms with CAD and vascular calcification, and none of these studies have been done in Latin America. Differences in allele frequencies and the linkage disequilibrium structure are also the result of interpopulation genetic differences. Therefore, genetic associations could differ between ethnic groups [19]. 

Considering the important role of PHACTR1 as a cellular process regulator, the aim of the study was to analyze the association of the *PHACTR1* rs2026458 and rs9349379 polymorphisms with premature CAD in a well-characterized Mexican clinical cohort.

## 2. Materials and Methods

This research analyzed the Genetics of Atherosclerotic Disease (GEA) cohort. The GEA was designed to investigate the genetic bases of premature CAD and subclinical atherosclerosis in relation with emerging risk factors in the Mexican population. The study complies with the Declaration of Helsinki and National normative, and additionally was approved by the Ethical and Research Committee from the National Institute of Cardiology “Ignacio Chavez” (INCICH) in September 2009 (approval code: 09-646). All participants signed an informed consent letter. 

### 2.1. Participant Selection and Clinical Evaluation

For this study, participants were enrolled from February 2010 to January 2012. Initially, the GEA cohort was divided in two groups, the first consisted of 994 patients with a history of premature CAD and the second were 1000 controls without a known history of CAD; then after coronary artery calcium (CAC) score assessment 297 controls were excluded due to the diagnosis of subclinical atherosclerosis (CAC score > 0). Consequently, the final number of controls was 703 participants. Additionally, all participants were Mexico City residents, who were at least the third generation born in Mexico, including their own. Furthermore, ancestry markers were analyzed in the participants. For the ancestry analysis, 265 markers defined according to the haplotype map project (HapMap) were determined, the percentage of Caucasian, native Indians and African ancestry was calculated using the admixture estimations calculated with the maximum likelihood method with the Leadmix software [20]. The results showed no significant difference between patients and control groups in relation to the frequency of native Indians genes (55.8% in patients and 54.0% in controls), Caucasian (34.3% in patients and 35.8% in controls) and Africans (9.8% in patients and 10.1% in controls). Therefore, the population was ethnically matched.

Patients with premature CAD were selected from the INCICH at Mexico City. The diagnosis was done with a previous history of myocardial infarction, angioplasty, coronary artery bypass graft (CABG) surgery, or coronary artery stenosis >50% on angiography, the age of diagnosis (before the age of 65 in women and 55 in men) according to the criteria of the American College of Cardiology (ACC) and the American Heart Association (AHA) [21].

Controls were volunteers recruited at the Blood Bank of the INCICH or individuals referred from Social Service Clinics, all controls had a negative family history of premature CAD (before the age of 65 in women and 55 in men) and no personal history of known cardiovascular disease. Patients and controls with previous history of liver, thyroid, malignant, or chronic renal disease, in addition those under corticosteroids treatment were excluded [22]. Physicians assessed all clinical variables and performed data collection. In addition, in all controls CAC score evaluation was performed using a computed tomography.

All participants answered standardized and validated questionnaires to obtain information on family and medical history, alcohol consumption, smoking history, cardiovascular risk factors, dietary habits and physical activity [23,24]. Positive alcohol use was considered when daily consumption exceeded more than 6 g of alcohol, in addition when participants smoked five or more cigarettes a day or had suspended this habit for less than a year, they were categorized as active smokers. During physical evaluation, anthropometric measures were recorded, height, weight, and waist circumference were measured and body mass index (BMI) was calculated. For other risk factors assessment, hypertension was defined as systolic blood pressure ≥ 140 mmHg, diastolic blood pressure ≥ 90 mmHg or as the current use of antihypertensive medication. Diabetes mellitus was considered according to the American Diabetes Association (ADA) criteria [25] and when participants were under glucose-lowering treatment due a previous diagnosis of diabetes mellitus. Furthermore, dyslipidemia was considered as total cholesterol ≥ 200 mg/dL and/or low-density lipoprotein cholesterol ≥ 130 mg/dL and/or triglycerides ≥ 150 mg/dL.

### 2.2. Coronary Artery Calcium (CAC) Score Evaluation

Computed tomography of the chest was performed using a 64-channel multi-detector helical computed tomography system (Somatom Cardiac Sensation, 64, Forcheim, Germany). Then, the scans were read by an experienced radiologist who assessed and quantified the CAC score, by using the Agatston method [26]. After CAC score evaluation, a total of 703 controls without evidence of subclinical atherosclerosis (CAC score = 0) were included in the analysis.

### 2.3. Genetic Analysis

Genomic DNA was extracted from leucocytes using the Lahiri and Nurnberger method [27]. The *PHACTR1* C > T rs2026458 (assay ID: C___1756602_10), and A > G rs9349379 (assay ID: C___1756707_10) SNPs were genotyped using 5 exonuclease TaqMan genotyping assays on an ABI Prism 7900HT Fast Real-Time PCR system (Applied Biosystems, Foster City, CA, USA) using the following conditions: Initial denaturation at 95 °C for 10 min, by 40 cycles of denaturation at 95 °C for 15 s and extension at 60 °C for 1 min. In addition, 200 samples were repeated and the results were consistent. Previously sequenced samples of the polymorphisms of all different genotypes were included as positive controls.

### 2.4. Statistical Analysis

We used SPSS version 23.0 (SPSS, Chicago, IL, USA) statistical software for Windows. Means ± standard deviation (SD) and frequencies of baseline characteristics were calculated. Statistical power to detect association with CAD was 0.80 as estimated with QUANTO software [28]. The Hardy-Weinberg equilibrium (HWE) was calculated using the Chi-square test. Comparison of numerical variables between both groups was performed using the Mann Whitney U. Categorical variables were analyzed with Chi-square test or Fisher’s exact test, as required, and presented as absolute frequencies and proportions. 

Inheritance hypothesis was tested according to five models: co-dominant, dominant, recessive, heterozygous, and additive. Multiple regression analysis was made to test association of polymorphisms with premature CAD adjusted for gender, age, hypertension, dyslipidemia, smoking habit, type 2 diabetes, and BMI. Pairwise linkage disequilibrium (D’, and r2) estimations between polymorphisms and haplotype reconstruction were performed with Haploview version 4.1 (Broad Institute, Cambridge, MA, USA). Additionally, a *p*-value of <0.05 was considered statistically significant.

### 2.5. Bioinformatics Prediction Analysis

We predicted the potential effect of the *PHACTR1* polymorphisms associated with premature CAD using bioinformatics tools, including FastSNP [29,30] and TFSEARCH database [31].

## 3. Results

### 3.1. Characteristics of the Study Population

Baseline characteristics of the premature CAD patients and controls included in the study are shown in Table 1. As expected, the prevalence of type 2 diabetes mellitus, a smoking habit, dyslipidemia and hypertension were higher in patients than in controls.

### 3.2. Allele and Genotype Frequencies

The allele and genotype frequencies of *PHACTR1* gene polymorphisms in premature CAD and controls are shown in Table 2. Observed and expected frequencies were in Hardy-Weinberg equilibrium.

A similar genotype distribution of the *PHACTR1* (rs2026458) polymorphism was observed in both study groups; however, a different genotype distribution of the *PHACTR1* (rs9349379) gene polymorphism was observed in patients and controls. Considering the minor allele frequency (MAF) as a reference in the construction of inheritance models, just the additive model was associated with the increased risk of developing premature CAD (odds ratio (OR) = 1.22, 95% confidence interval (CI) = 1.03–1.46, *p*-value (*p*) = 0.024) (Table 2). This model was adjusted for age, gender, hypertension, BMI, dyslipidemia, smoking habit, and type 2 diabetes mellitus.

### 3.3. SNP Function Prediction

Based on SNP functional prediction software, only the rs9349379 polymorphism studied seems to be functional. This polymorphism affects the DNA binding site for myocyte enhancer factor 2 (MEF2).

## 4. Discussion

Currently, the role of *PHACTR1* in CAD pathogenesis is still under study. *PHACTR1* is expressed in the brain, kidney, testes, lung, heart and endothelial cells. In endothelial cells, PHACTRs interact as mediators of PP1, an enzyme that regulates endothelial nitric oxide and has been proved to be an important modulator in the pathogenesis of cardiovascular disease and endothelial dysfunction [32,33]. Further, *PHACTR1* gene polymorphisms have been associated with several diseases, including hypertension, diabetes mellitus, cervical artery dissection and myocardial infarction [14,34,35,36].

PHACTR1 has been recently implicated in molecular pathways in all cardiovascular system components. Indeed, Kelloniemi et al. [8] described the role of PHACTR1 in the α-actin of the cytoskeleton of rat myocardiocytes.

In the present work, two *PHACTR1* gene polymorphisms (rs2026458 and rs9349379) were analyzed, to establish their role as susceptibility factors for premature CAD in the Mexican population. Herein, we established that the rs9349379 polymorphism was associated with an increased risk of premature CAD. Similar results were published by Beaudoin et al. [16], who demonstrated the association between the rs9349379 polymorphism and the *PHACTR1* expression levels in 25 human right coronary arteries from patients undergoing heart transplantation (*p* = 0.018). On the other hand, the functional prediction analysis used in our study predicted that the rs9349379 polymorphism has a potential functional effect; this variant produces a binding site for MEF2. This transcriptional factor plays a fundamental role in the differentiation, morphogenesis, proliferation, apoptosis, and survival of a wide spectrum of cell types. In addition, it acts as a mediator of epigenetic mechanisms that include changes in chromatin, and the modulation of microRNAs [37]. This result is similar to that reported by Beaudoin et al. [16], who performed electrophoretic mobility shift assays (EMSA) with HUVEC nuclear extracts and demonstrated the interaction between the rs9349379 polymorphism and the MEF2 factor. A GWAS performed in a Lebanese study recently confirmed the association of the *PHACTR1* (rs9349379) gene polymorphism with coronary artery stenosis [5]. We agree with this report, since our data confirmed that the rs9349379 polymorphism is associated with premature CAD in a Mexican population. Moreover, O’Donnell et al. [6] identified common genetic variants in the *PHACTR1* gene (rs9349379 and rs2026458 polymorphisms) associated with coronary artery calcification during follow-up in myocardial infarction. In our study, the distribution of the rs2026458 polymorphism was similar in patients with CAD and the controls; also the minor allelic frequency of rs2026458 was in accordance with the report by O’Donell et al. (0.46) who performed a GWAS for coronary artery calcification [6]. These discrepancies of our results with those reported by other authors may be related to differences in the genetic background of the studied populations. The Mexican population is the result of a unique mixture between the immigrants that arrived to the Mexican territory during the 16th century and the autochthonous population. Thus, from the genetic standpoint, comparing the Mexican population, which is actually the result of the genetic contributions of different ethnic groups, might lead to the failure to replicate genetic association findings from this particular study in other populations. On the other hand, it is essential to consider the effect of the environmental factors, which include pollution, lifestyle, stress, and fast food consumption, among others, in complex and polygenic diseases such as CAD. These discrepancies may be related to differences in the genetic background in the studied population; genetic heterogeneity is a well-recognized reason for the failure to replicate genetic association findings. In addition, the rs9349379 polymorphism is a tagSNP according the HapMap in the Caucasian population (Utah residents with northern and western European ancestry), Han Chinese, Japanese, and Los Angeles Mexican population [38]. Furthermore, this is the first reported article focused on the native Mexican population. Nonetheless, considering that predominantly *PHACTR1* GWASs are mainly performed in specific populations in the Middle East, Asia and Europe, these results do not necessarily provide information about the Mexican population. This is due to the particular ancestry of the Mexican population, which is the result of the mixture of the native inhabitants of the region, Europeans (60%–64%), Amerindians (25%–21%), and Africans (15%) [39,40]. 

Therefore, *PHACTR1* is associated with metabolic pathways that have a significant influence on the homeostasis of the cardiovascular system. Although some of the metabolic pathways in which this gene interacts are well identified, others have not been completely clarified yet. Consequently, further functional analyses in cellular and animal models of the *PHACTR1* gene polymorphisms will allow a deeper understanding of CAD pathophysiology. Moreover, these polymorphisms could also be used as markers for cardiovascular disease in the Mexican population. Additionally, this could decrease the rate of drug-related adverse effects in different ethnic groups and lead to a better quality of life of the general population. Thus, it is plausible to expect in the future that physicians will have more and improved therapeutic options for the treatments of cardiovascular disease, a significant public health problem which is currently the most important cause of death worldwide and a cause of substantial disability [41,42].

Study limitations need to be addressed. In this report, only two gene polymorphisms were analyzed, and there are important differences between both groups in relation to gender, age and comorbidities. In addition, there is a survival bias in which only patients who survived a myocardial infarction event were included in the patient group. Additionally, we expect that in the future, other authors will be able to replicate our results, and further studies will analyze the association of other *PHACTR1* gene polymorphisms.

## 5. Conclusions

In summary, CAD is an important public health problem in Mexico; this case-control study provides information in relation to the association of the rs9349379 polymorphism of the *PHACTR1* gene with premature CAD in the Mexican population. However, the findings should also be taken with caution, as no follow-up was performed. Therefore, prospective studies with long-term follow-up, performed in other populations, are needed to better understand the contribution of the *PHACTR1* gene in CAD.

## Figures and Tables

**Table 1:** Baseline clinical characteristics in patients with premature CAD and controls.

Parameter		Premature CAD (*n* = 994)	Control (*n* = 703) (%)	*p*-Value
Gender n (%)	Male	792 (79.7)	271 (38.5)	<0.0001 *
	Female	202 (20.3)	432 (61.5)	
Age (years)		53.47 ± 7.33	52.33 ± 9.06	0.001 *
Body Mass Index (Kg/m^2^)		28. 84 ± 4.31	28.31 ± 4.58	0.002 *
Type 2 diabetes n (%)		340 (34.2)	68 (9.7)	<0.0001 *
Smoking habit n (%)		750 (75.5)	377 (53.6)	<0.0001 *
Dyslipidemia n (%)		546 (54.9)	309 (44)	<0.0001 *
Hypertension n (%)		697 (70.1)	207 (29.4)	<0.0001 *
Use of alcohol n (%)		814 (81.9)	567 (80.7)	0.280

The variables are expressed as the mean ± standard deviation (SD); BMI, body mass index; CAD, coronary artery disease; * Considered statistically significant when *p* < 0.05.

**Table 2:** Association of the *PHACTR1* polymorphisms with premature CAD.

Genotype Frequency n (%)				MAF	Model	OR (95%CI)	*p*-Value
***PHCTR1*** **A > G** **rs9349379**	***AA***	***AG***	***GG***				
Premature CAD (*n* = 994)	349 (0.351)	479 (0.482)	166 (0.167)	0.410	Co-dominant	1.51 (1.05–2.17)	0.077
					Dominant	1.27 (0.99–1.63)	0.061
					Recessive	1.36 (0.98–1.90)	0.068
Controls (*n* = 703)	285 (0.405)	320 (0.455)	98 (0.139)	0.370	Heterozygous	1.06 (0.83–1.35)	0.640
					Additive	1.22 (1.03–1.46)	0.024 *
***PHACTR1*** **C > T** **rs2026458**	***CC***	***CT***	***TT***				
Premature CAD (*n* = 994)	348 (0.350)	490 (0.493)	156 (0.157)	0.400	Co-dominant	0.87 (0.60–1.26)	0.740
					Dominant	0.91 (0.71–1.18)	0.490
Controls (*n* = 703)	247 (0.351)	337 (0.479)	119 (0.169)	0.410	Recessive	0.91 (0.66–1.26)	0.570
					Heterozygous	0.97 (0.76–1.24)	0.810
					Additive	0.93 (0.78–1.11)	0.440

The models were adjusted for age, gender, body mass index, type 2 diabetes, smoking habit, dyslipidemia, and hypertension. CAD, coronary artery disease; MAF, minor allele frequency; OR, odds ratio; 95%CI, confidence interval. * Consider statistical significant when *p* < 0.05.
